# Flexible Methyl Cellulose/Polyaniline/Silver Composite Films with Enhanced Linear and Nonlinear Optical Properties

**DOI:** 10.3390/polym13081225

**Published:** 2021-04-10

**Authors:** Ali Atta, Mostufa M. Abdelhamied, Ahmed M. Abdelreheem, Mohamed R. Berber

**Affiliations:** 1 Physics Department, College of Science, Jouf University, Sakaka P.O. Box: 2014, Saudi Arabia; aamahmad@ju.edu.sa; 2 Radiation Physics Department, National Center for Radiation Research and Technology (NCRRT), Atomic Energy Authority (AEA), Cairo P.O. Box: 29, Egypt; mostufa.abdelhamied@eaea.org.eg (M.M.A.); ahmed.abdelreheem2009@eaea.org.eg (A.M.A.); 3 Chemistry Department, College of Science, Jouf University, Sakaka P.O. Box: 2014, Saudi Arabia; 4 Department of Chemistry, Faculty of Science, Tanta University, Tanta 31527, Egypt

**Keywords:** methyl cellulose, polyaniline, silver nanoparticles, optical properties

## Abstract

In order to potentiate implementations in optical energy applications, flexible polymer composite films comprising methyl cellulose (MC), polyaniline (PANI) and silver nanoparticles (AgNPs) were successfully fabricated through a cast preparation method. The composite structure of the fabricated film was confirmed by X-ray diffraction and infrared spectroscopy, indicating a successful incorporation of AgNPs into the MC/PANI blend. The scanning electron microscope (SEM) images have indicated a homogenous loading and dispersion of AgNPs into the MC/PANI blend. The optical parameters such as band gap (Eg), absorption edge (Ed), number of carbon cluster (N) and Urbach energy (Eu) of pure MC polymer, MC/PANI blend and MC/PANI/Ag films were determined using the UV optical absorbance. The effects of AgNPs and PANI on MC polymer linear optical (LO) and nonlinear optical (NLO) parameters including reflection extinction coefficient, refractive index, dielectric constant, nonlinear refractive index, and nonlinear susceptibility are studied. The results showed a decrease in the band gap of MC/PANI/AgNPs compared to the pure MC film. Meanwhile, the estimated carbon cluster number enhanced with the incorporation of the AgNPs. The inclusion of AgNPs and PANI has enhanced the optical properties of the MC polymer, providing a new composite suitable for energy conversion systems, solar cells, biosensors, and nonlinear optical applications.

## 1. Introduction

Flexible composite materials have been receiving a great deal of attention in optical energy applications due to their remarkable electrical, thermal, mechanical, dielectric and optical properties versus the other traditional materials [1,2]. Currently, polymer composites are of high interest in different energy applications because of their pliable characteristics and their easy to use [3]. Huge attention has been given to reveal the optical properties of polymers, and to potentiate their implementations in optical energy applications [4].

Among the various polymers used in optical energy applications, is the polyaniline (PANI) which is widely studied as a nonlinear optical (NLO) material because of its UV radiation resistance, ultrafast response, steady electrical conductivity, flexibility and easy relative processing [5]. However, PANI has limited mechanical properties, fusibility, and solubility [6]. Thus, it has to be modified with other materials to improve these limitations and to enhance its uses, especially in energy applications. On the other hand, methyl cellulose (MC) polymer is a water soluble polymer with a semi-crystalline structure. It carries high mechanical properties, a unique chemical stability, and a perfect optical behavior [7]. Hence, the composite of MC and PANI is envisioned to provide a material with remarkable optical properties.

The incorporation of inorganic fillers into the polymer matrix is of much interest, because such inorganic polymer composites have paved the road to modify the polymer composite properties, providing additional and remarkable optical features [8]. Conducting fillers such as silver nanoparticles (AgNPs) have showed many advantages in various applications (e.g., conductive inks, photonics and electronic chips, sensors, batteries and solar cells) because of their localized surface plasmon resonance properties, and their special optical, electrical, and thermal properties which decreases the optical band gap. The properties have made AgNPs attractive materials for surface-enhanced Raman scattering substrates (SERS) and optical sensor applications [9,10]. AgNPs can be synthesized by different methods including chemical reduction, which is one of the most widely used methods for preparing AgNPs in water or organic solvents, thermal decomposition, electrochemical reduction, photo reduction, and biosynthesis [11,12]. Accordingly, the incorporation of AgNPs into the MC/PANI polymer matrix is a topic of interest.

In the current study, we aim to prepare a flexible polymeric composite film of AgNPs and the MC/PANI polymer matrix, studying the possibility of enhancing the optical properties of MC. We characterize the prepared polymers and the composites by different spectroscopic techniques including molecular weight determination, XRD, FT-IR, and SEM. The linear/nonlinear optical parameters (extinction coefficient, refractive index, reflectance, first order linear optical susceptibility, real and imaginary dielectric constant, and third order nonlinear optical susceptibility) of MC/PANI/AgNPs composite films will be determined by using the UV–Vis spectroscopy. 

## 2. Materials and Methods

### 2.1. Materials

Methyl cellulose (MC; MW 658.7 g/mol) was purchased from Sigma–Aldrich (Jeddah, Saudi Arabia); hydrochloric acid (35%), and ammonium peroxidisulfate (grade, 99%), were purchased from Merck (Darmstadt, Germany); aniline monomer (C6H5NH2) was purchased from Oxford Lab Chemistry (Maharashtra, India), and AgNO3 was purchased from Nice Chemicals (Kerala, India).

### 2.2. Synthesis of PANI

PANI was prepared by an oxidation polymerization process of aniline in hydrochloric acid using ammonium peroxide-sulfate as an oxidative agent [13]. Firstly, aniline monomer was vacuum distilled before use for the polymerization process. Then, in a precooled ice bath (temperature <0 °C) containing 100 mL of 1 M, a few grams of the distilled aniline monomer were added and dissolved upon sonication. Subsequently, a solution of the ammonium persulphate initiator was added dropwise over a period of 30 min. The reaction flak was then vigorously stirred for 2 h at 0 °C to complete the polymerization process. The color change from colorless to dark green indicated the synthesis of PANI. The formed PANI was then vacuum filtered, rinsed by 1M HCl until a colorless filtrate was obtained, washed by distilled water, and finally vacuum dried at 70 °C overnight.

### 2.3. Molecular Weight (MW) Determination of PANI

The MW of PANI was determined via the inherent viscosity measurement at 30 °C using an Ubbelohde viscometer. For that purpose, four different concentrations (namely; 0.5, 0.25, 0.2, and 0.1 g/dL in concentrated sulphoric acid) were prepared. The obtained inherent viscosity was used to estimate the MW of PANI using the Mark–Houwink–Sakurada equation [14]. The average MW of PANI was 29 KD.

### 2.4. Preparation of MC/PANI/AgNPs Composite Film 

PANI was prepared by an oxidation polymerization process of aniline in hydrochloric acid using ammonium peroxide-sulfate as an oxidative agent. For the synthesis of the MC/PANI blend, 1.0 g of MC was dissolved in 50 mL of distilled water at room temperature. Then, different amounts of 1.0 wt% and 3.0 wt% of PANI were added separately to the MC solution. The resultant mixture was stirred by an ultrasonic probe at room temperature until a homogenous solution was obtained. 

Regarding the preparation of MC/PANI/AgNPs composite films, AgNO_3_ powder was added with different concentrations that were added to the MC/PANI blend as follows. In two conical flasks containing the similar amounts of MC/PANI blends, 0.5 wt% and 1.0 wt% of AgNO_3_ were added over a period of 30 min under continuous stirring. Then, 30 mL of the reducing agent NaBH_4_ (2mM) was added dropwise and separately to each flask to reduce the Ag ions to metallic AgNPs. Then, the composite mixture was stirred over a period of 60 min. Subsequently, the obtained mixture was poured onto a Petri dish to cast a film. The cast films were coded MC film, MC/1%PANI, MC/3%PANI, MC/3%PANI/0.5AgNPs, and MC/3%PANI/1AgNPs.

### 2.5. Characterization Techniques

The structural properties of the MC film, MC/PANI blend and the MC/PANI/Ag composite films were investigated by using X-ray powder diffraction (Shimadzu XRD-6000). The chemical bonds and functional groups of the prepared films were identified using FTIR spectroscopy (ATI Mattson, Genesis series, Unicam, UK). A scanning electron microscope (SEM) JEOL, Tokyo, Japan was used to investigate the morphological changes of the formed films. The optical properties of the prepared films were investigated by a UV–Vis spectrometer (JascoV-670 spectrophotometer, Tokyo, Japan) in the wavelength 200 nm to 1100 nm. The absorbance (A) and the reflectance (R) were directly measured by using the spectrophotometer. The R and A data were used to determine the optical constants, i.e., the absorption coefficient (α), the refractive index (n) and the extinction coefficient (k). The Wemple and DiDomenico (WDD) single-effective oscillator model has been used to determine inter-band optical transitions and the dependence of optical dispersion on the refractive index. In this model, frequency-dependent dielectric constants were used to define the oscillator energy (E_o_) and dispersion energy (E_d_) parameters. The WDD model was used to fit the experimental results as it provides an intuitive physical interpretation of the measured quantities.

## 3. Results and Discussion

### 3.1. Structural Investigation of the Synthesized Films

**Figure 1:**
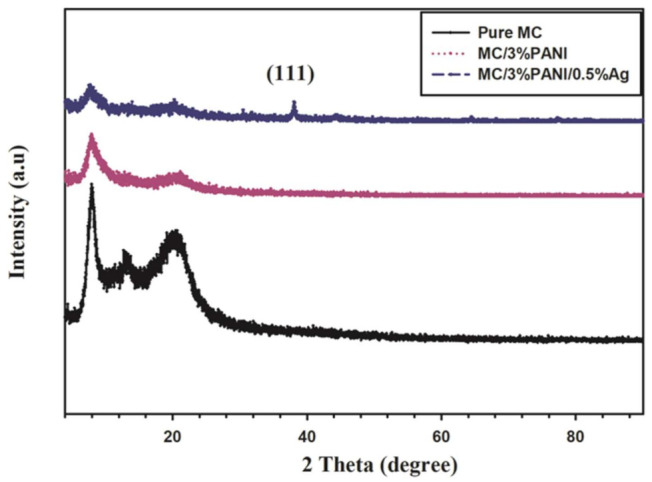
XRD patterns of pristine methyl cellulose (MC), MC/3% polyaniline (PANI) and MC/3%PANI/0.5Ag films.

The XRD spectrum of the pristine MC, PANI/MC blend, and the MC/PANI/AgNPs films are displayed in Figure 1. The spectrum of MC indicated two diffraction peaks at 8.1° and 20.7°, characteristic for MC with a partial crystalline structure. The area under the peaks 8.1° and 20.7° of MC was 1104 and 2353, respectively. The XRD pattern for the MC/3%PANI film became more amorphous with the near disappearance of the MC diffraction peak at 20.7°. The area under the peak 8.1° of MC changed from 1104 to 4066, confirming the amorphous structure of MC obtained after the addition of PANI. This reduction in the MC crystallinity suggests a good interaction of the chains of MC and PANI polymers [15]. The XRD of MC/3%PANI/0.5%AgNPs (blue curve) showed a new peak at 38.6°, characteristic for the (111) crystallographic plane of the face-concentrated-cubic AgNPs. The expansion of the full width half maximum (FWHM) of the 8.1° diffraction peak (from 1.30 for pure MC to 1.85 for MC/3% PANI blend) indicates a diffusability of the PANI into the MC matrix. The average crystallite size (D) of AgNPs determined from the Scherrer equation [16] was 13.5 nm.

**Figure 2:**
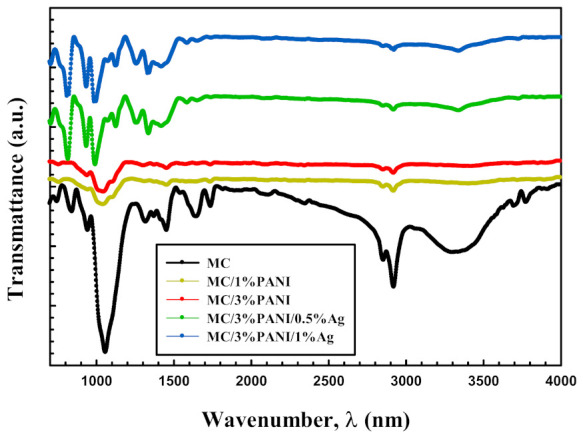
IR spectra of pristine MC, MC/1%PANI, MC/3%PANI, MC/3%PANI/0.5%Ag nanoparticles (NPs) and MC/3%PANI/1AgNPs films.

Furthermore, the IR data displayed in Figure 2 confirmed the structure of the synthesized films. Specifically, the black IR spectrum of Figure 2 showed the characteristic absorption bands C–O–C stretching vibration, C–H stretching vibration, and O–H stretching vibration of MC at 1110 cm^−1^, 2900 cm^−1^ and 3400 cm^−1^, respectively [15]. After the addition of PANI (yellow and red spectrum), the intensity of the absorption bands of MC lowered, especially the OH stretching vibration band. This lowering of the beaks intensity suggested a breakage of the hydrogen bonding network of MC and the penetration of PANI between the chains of MC. In addition, the characteristic absorption bands of PANI, in particular, the C–N and C = N stretching vibrations newly emerged at 1250 cm^−1^ and 1550 cm^−1^, respectively [16]. The addition of AgNPs (green and blue spectra) showed no change in the spectra of MC/PANI blends. Only new bands characteristic for the metal particles were observed at 750 and 820 cm^−1^ in the spectrum of the MC/PANI/AgNPs composite [17]. 

**Figure 3:**
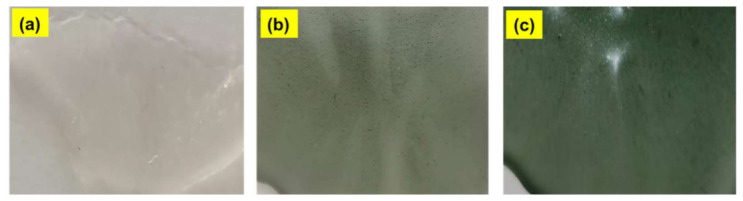
Photo images of: (**a**) pristine MC, (**b**) MC/1%PANI, and (**c**) MC/3%PANI films.

Figure 3a–e shows the photograph images of pure MC (image a), MC/1%PANI (image b), and MC/3%PANI (image c) films. As can be seen, the color of the MC film gradually changed from white to light green, and then dark green after the addition of 1 wt% and 3 wt% of PANI, respectively [18].

**Figure 4:**
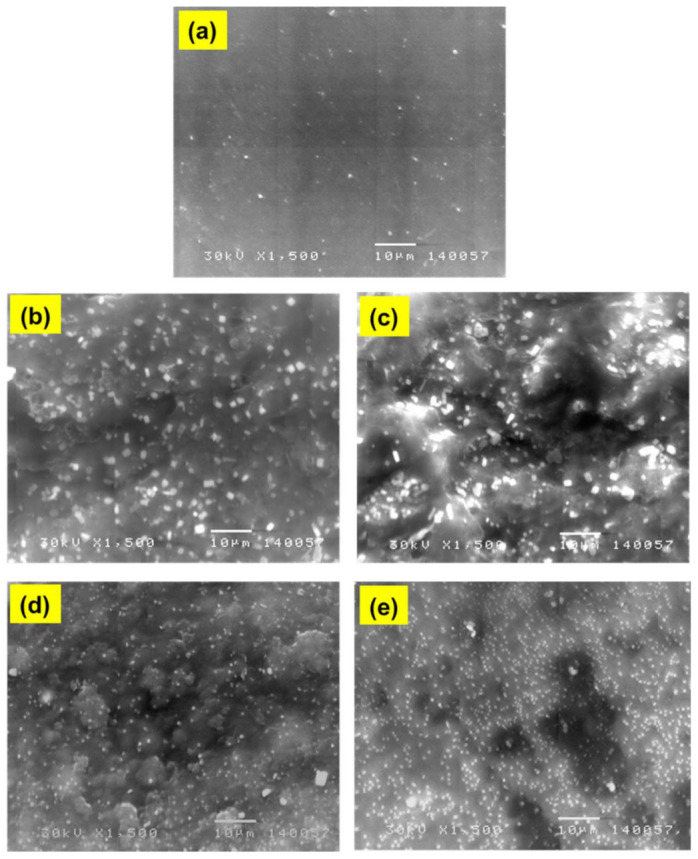
SEM images of: (**a**) pure MC film, (**b**) MC/1%PANI, (**c**) MC/3%PANI, (**d**) MC/3%PANI/0.5%AgNPs, and (**e**) MC/3%PANI/1%AgNPs films.

SEM was employed to study the surface morphology of the fabricated films, and to show the distribution of the AgNPs into the formed films. The collected SEM images of Figure 4 provided useful information for understanding the structural and the changes of the optical properties of the films under the influence of PANI and AgNPs additions. As can be seen, the SEM image of pure MC film (image a) shows a smooth and uniform surface, while the SEM images of MC/1%PANI and MC/3%PANI films (images b and c) display rough surfaces with the emerge of some small aggregates corresponding to PANI [19,20,21]. The SEM images (d and e) show a good distribution of the AgNPs into the MC/PANI films, indicating a sufficient interfacial interaction to MC/PANI blend matrix and the AgNPs [22].

### 3.2. Optical Properties of the Synthesized Films

**Figure 5:**
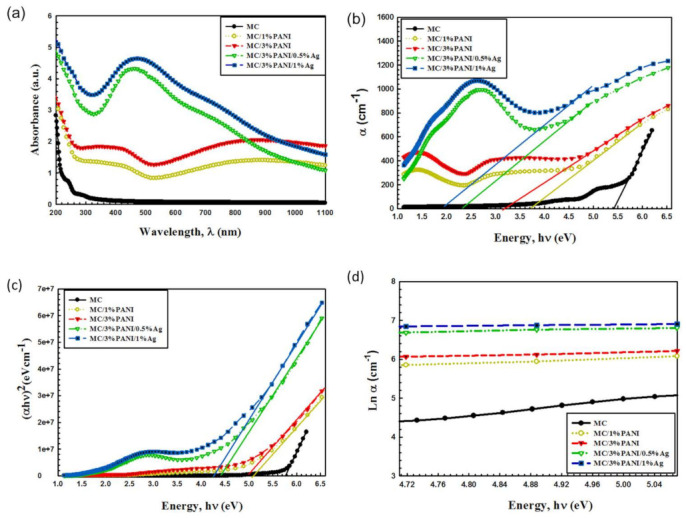
(**a**) UV–Vis absorption spectra, (**b**) absorption coefficient α, (**c**) the relation between (αhν)^2^ against photon energy (hν), (**d**) absorption coefficient *lnα* versus photon energy for pristine MC, MC/1%PANI, MC/3%PANI, MC/3%PANI/0.5AgNPs and MC/3%PANI/1%AgNPs films.

The UV–Vis measurements of MC, MC/PANI, and MC/PANI/AgNPs films are displayed in Figure 5a. As observed, the MC absorption peak increased by the addition of PANI. In addition, the two peaks of MC and PANI overlapped as a result of the blending process. After the addition of AgNPs, a new adsorption peak characteristic for the surface plasmon resonance (SPR) of the electrons of AgNPs was observed. From the FWHM (∆*E*_1/2_) of the SPR band, the radius (r) of AgNPs has been estimated assuming free particles behavior of conduction electrons, using Equation (1) [23]:

(1) $$r=\;\frac{h{\;v}_{f}}{∆E_{1/2}}$$

where *h* is the Planck’s constant, and $v_{f}$ (1.39 × 106 m/s) is the Fermi velocity of electrons. The AgNPs average particle size was found to be 10 nm, which is closer to the crystallite size value calculated from XRD. 

The absorption coefficient α (ν) is estimated using Equation (2) [24].

(2) $$\alpha \left(\nu \right)=\frac{2.303\;A}{d}$$

where A indicates the optical absorbance and d is the film thickness. The absorption optical coefficients with photon energy of pristine MC, MC/1%PANI, MC/3%PANI, MC/3%PANI/0.5AgNPs, and MC/3%PANI/1AgNPs films are shown in Figure 5b. The absorption edge (*E*_d_) value was determined from extrapolating the linear part of 𝛼 against applied hν curves at absorption zero value [24]. As shown, the absorption edge *E*_d_ decreased by the addition of AgNPs and PANI to MC polymer (see the details of Table 1). The *E*_d_ decreased from 5.42 eV for pristine MC to 3.14 eV for MC/3%PANI and reduced to 1.93 eV for MC/3%PANI/1%AgNPs. This reduction in *E*_d_ has been related to the changes in the number of the electrons and the holes in the conduction bands and the valence bands [25]. Another indicator for the shifting of the absorption edge was the structural modifications in MC matrix, which has been determined by the XRD results and the molecular interactions of MC polymer chains and the AgNPs. Specifically, the shift in the optical absorption edge reflects the electronic conjugation between the AgNPs and the PANI beside the increase in the degree of disorder for those composite films [26].

The energy band gap (*E_g_*) of all films were determined from the intercept of the extra plotted linear portion (α hν)^2^ versus hν as shown in Figure 5c, which pursues the Tauc method (Equation (3)) [27]:

(3) $$\alpha h\nu =\;B{\left(\mathrm{hv}\;-\;E_{g}\right)}^{n}$$

where B is the width parameter of the absorption edge, hν is the incident photon energy calculated from hν = 1240/λ, and n is factor takes 3/2 or 1/2 for direct transitions and 2 or 3 for indirect transitions relaying on the forbidden or allowed transition, respectively. The determined values of direct *E_g_* are listed in Table 1. As observed, *E_g_* decreased from 5.76 eV for pristine MC, to 5.01eV for MC/3%PANI and reduced to reach 4.27eV for MC/3%PANI/1%AgNPs. The reduction in MC optical gap was due to the variations of polymer disorder [28]. In addition, the defects induced by the localized states in the band gap was the main reason for the reducing energy band of the polymer. Furthermore, the reduction in optical band gap was related to the charge transfer complexes’ (CTCs’) formation due to the trap levels between LUMO and HOMO bands of the MC polymer [29], which enhanced the lower transitions’ energy. This successfully implies the miscibility of AgNPs and MC/PANI blend chains and strongly supports the XRD results.

For determining the band tail that refers to the width of localized states, the absorption coefficient *α* (*v*) near the band edge as exponential dependence of photon energy (hv) has been determined from the Urbach relationship (Equation (4)) [30]:

(4) αν=αo ehvEe 

The Urbach tail *E_e_* value, recorded in Table 1, has been determined by the reciprocal of the slope of the linear portion of the curves shown in Figure 5d. As observed, *E_e_* enhanced from 0.51 eV for pristine MC to 1.52 eV for MC/3%PANI and increased to reach 2.02 eV for MC/3% PANI/1%AgNPs. The band tail E_e_ values inversely changed with the band gap *E_g_* values. This was a result of the disorder induced in the MC polymer chain after the incorporation of the AgNPs that induced interaction changes in MC/PANI polymer blend [31]. In other words, the changes in the Urbach tail *E_e_* was a result of the defects in the MC chains after the addition of PANI and AgNPs. Specifically, the AgNPs and PANI led to the redistribution from the band states, permitting additional tail to tail transitions [32]. 

The carbon cluster number (*N*) was estimated from the optical gap *E_g_* using Equation (5) [33]:

(5) $$E_{g}=34.4/\sqrt{N}$$

The estimated *N* value (see Table 1) of pristine MC was 36. After the addition of PANI, it increased to be 47, and has increased to reach 65 for MC/3%PANI/1%AgNPs film. This enhancement of *N* number was a result of the conjugation of monomer units in the MC polymer matrix after the addition of PANI and AgNPs in the MC polymer. The enhancement of the *N* values has been attributed to the amounts of the conducting PANI and the AgNPs, where the higher their content in the host MC matrix, the more defects are introduced, causing additional low energy states and hence a decrease in the band gap is observed, leading to an improvement in the *N* value.

**Figure 6:**
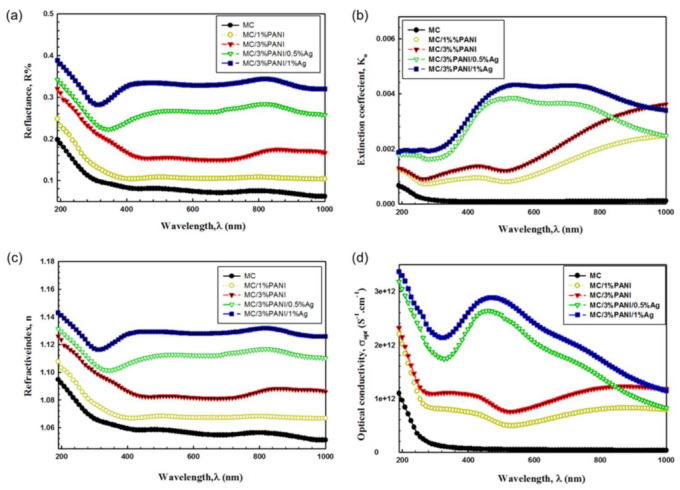
(**a**) the reflectance R, (**b**) the extinction coefficient k, (**c**) the refractive index n, and (**d**) the optical conductivity σ opt; as a function of wavelength λ for pristine MC, MC/1%PANI, MC/3%PANI, MC/3%PANI/0.5%AgNPs and MC/3%PANI/1%AgNPs films.

Figure 6a shows the relation of the reflectance (R) versus the wavelength (λ) for pristine MC, MC/PANI and MC/PANI/AgNPs films. As noticed, the reflectance R value was enhanced by the addition of PANI and AgNPs. This increase in reflectance R is attributed to a reduction in incident light scattering, which reflects the changes of the disorder degree of the polymer chain [34]. This propagation of light through MC/PANI/AgNPs composite-based materials relies on the functional groups of PANI as well as the nature of AgNPs. Furthermore, the behavior changes of the reflectance R with PANI and AgNPs is attributed to the increase in the packing density of PANI and the AgNPs content in the composite. In other words, the changes of the R value have reflected the effects of both PANI and AgNPs for modifying the electronic structure of the MC polymer chain [35].

The extinction coefficient (*K*), which is an indication of the debility of alterations of absorption when the electromagnetic waves passthrough the composite, was calculated using Equation (6) [36].

(6) $$K=\frac{\alpha \lambda }{4\pi }$$

The extinction coefficient k is plotted in relation to the wavelength, as shown in Figure 6b. As seen, k displayed high values at the longest wavelengths. This behavior is ascribed to the high absorption coefficient in this region. On other hand, the extinction coefficient k was enhanced by the addition of PANI and AgNPs to the MC polymer chain. This is because PANI and AgNPs induce the modification of the polymer structure. Another factor for the enhanced *k* was a result of the surface SPR of drugged AgNPs in MC polymer. Additionally, the extinction coefficient K of MC/3%PANI/0.5%AgNPs observed at λ of 490 nm shifted to λ of 520 nm for MC/3%PANI/1%AgNPs film. This shift in wavelength with increasing AgNPs was a result of the new levels in the optical band gap that led to increased access of electrons from the valence band to the conduction band [37]. Furthermore, the higher value of the extinction coefficient of MC/PANI/1%AgNPs showed increased scattering of light compared to MC/PANI/0.5%AgNPs. This increase in the extinction coefficient of MC/PANI/1%AgNPs was a result of the enhanced interactions of AgNPs and PANI [38].

The refractive index (*n*), which indicates the electronic polarizability of the ions into the material, is considered as one of the most important parameters in optical physics for developing optical transmission and spectral dispersion instruments. Accordingly and in order to deduce the effects of the AgNPs interaction with the MC polymer matrix, the optical dielectric properties and refractive index were investigated. Figure 6c shows the changes of n as a function of wavelength λ for pristine MC, MC/1%PANI, MC/3%PANI, MC/3%PANI/0.5%AgNPs and MC/3%PANI/1%AgNPs films. The refractive index n is thus calculated from Equation (7) [39]:

(7) $$n=\frac{\left(1+R\right)}{\left(1-R\right)}+\sqrt{\frac{4R}{{\left(1-R\right)}^{2}}}-K^{2}$$

As can been seen, the n is first decreased by a wavelength increase for all the films, as shown in Figure 6c. Next, a steady rate is observed at the higher wavelengths, demonstrating the normal light dispersion. Furthermore, the refractive index n was enhanced by the addition of PANI and AgNPs the MC polymer chains. This is because of localized hesitance of charged particles in the conducting materials. Moreover, the incorporation of AgNPs and PANI into the MC polymer has improved the film density to make it compact and hard [40]. In other words, the increase in the refractive index was a result of the incorporation of both PANI and AgNPs and their interaction with the chains of MC, which led to an enhancement in the density of the composite films.

The optical conductivity (*σ_opt_*) which demonstrates the electrical conductivity that was produced as a result of the charge carrier’s transfer due to the variation in the electric field of the fallen electromagnetic waves was determined from the absorption coefficient (α) using Equation (8) [39]:

(8) $$\sigma_{opt}=\frac{\alpha nc}{4\pi }$$

where *c* is the speed of light and *n* is the refractive index. The change of *σ_opt_* with photon energy is shown in Figure 6d. At high photon energy, we can see the enhancement of the *σ_opt_* of films where the high absorbance value in that region led to an increase in the charge transfer excitations. Notably, the *σ_opt_* improved with the increase in both the PANI and the AgNPs. This improvement was correlated to the increase in the localized stage densities in the band structure. In other words, it can be said that AgNPs modified the functional composite structure because of the intra-band transitions of conduction electrons [41]. The characteristics of the inter-band optical transitions changed due to the variations in structural properties, which have a significant influence on electrons/holes, generating specific dispersion parameters [42]. Therefore, the optical dielectric properties and the optical conductivity data reflect the importance of the MC/PANI/Ag composite films for optical energy devices.

In order to highlight the optical properties of the newly fabricated MC/PANI/AgNPs composite films, the characteristic dielectric properties were measured. The dielectric properties are envisioned to give an understanding of the polarization of the MC/PANI/AgNPs composite films in the optical devices. In particular, we measured the imaginary *ε_i_* and real *ε_r_* dielectric parts which are the most basic material properties that demonstrate the light dispersion of the films. *ε_i_* and *ε_r_* were determined from Equation (9) [43]:

(9) $$\varepsilon =\varepsilon_{r}+i\varepsilon_{i}$$

The real part *ε_r_* was estimated from of the extinction coefficient *k* and the refractive index *n* data using Equation (10) [43]:

(10) $$\varepsilon_{r}=n^{2}-K^{2}\;(10)$$

**Figure 7:**
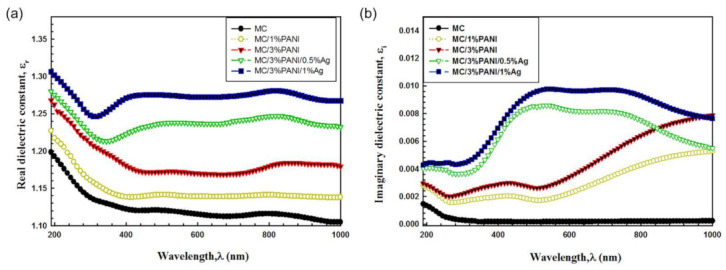
(**a**) Real dielectric constant ε_r_ and (**b**) imaginary dielectric constant ε_i_ as a function of wavelength λ for pristine MC, MC/1%PANI, MC/3%PANI, MC/3%PANI/0.5%AgNPs and MC/3%PANI/1%AgNPs films.

The variation of the real dielectric constant *ε_r_* versus the wavelength λ for pristine MC, MC/1%PANI, MC/3%PANI, MC/3%PANI/0.5%AgNPs and MC/3%PANI/1%AgNPs films are shown in Figure 7a. As can be seen, the real dielectric constant *ε_r_* part enhanced with the increase of both the AgNPs and PANI contents, indicating a good dispersion of PANI and AgNPs into the MC polymer chain, because of the induced structure changes of Ag and PANI in the polymer matrix. By changing the silver content from 0.5% to 1% in the MC/3%PANI blend, the motion of charge carriers and localized charged particles fluctuations induced modifications in the optical dielectric dispersion properties, indicating flexible films for optical energy applications. 

The imaginary part *ε_i_*, which demonstrates the absorbed energy of the electric field as a result of dipole moment motion, is given by the following equation (Equation (11)) [43]:

(11) $$\varepsilon_{i}=2\;n\;k$$

The variation of *ε_i_* constant with the photon energy (hν) of MC, MC/1%PANI, MC/3%PANI, MC/3%PANI/0.5%AgNPs and MC/3%PANI/1%AgNPs films is shown in Figure 7b. As noticed, the behavior of *ε_i_* is similar to the behavior of *k*. Probably, this is because the refractive index is very tiny. The imaginary dielectric constant was improved by the addition of PANI and AgNPs into the MC polymer and the MC/PANI blend. This is because the formed defects induced charge transfer reactions between the MC chains and the AgNPs dopant [44].

**Figure 8:**
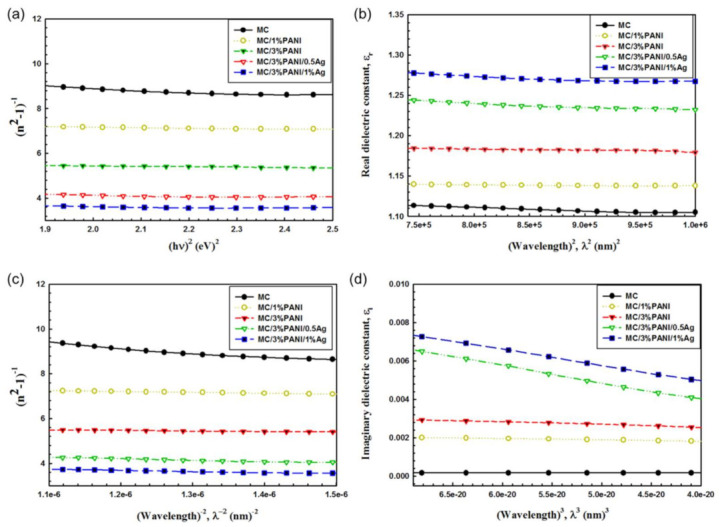
The relations between: (**a**) (*n*^2^ − 1)^−1^ and (hυ)^2^, (**b**) dielectric constant ε_r_ and λ^2^, (**c**) (*n*^2^−1)^−1^ and λ^−2^ and (**d**) imaginary dielectric constant ε_i_ and λ^3^; for pristine MC, MC/1%PANI, MC/3%PANI, MC/3%PANI/0.5%AgNPs and MC/3%PANI/1%AgNPs films.

The refractive index values can be modeled according to the single oscillator paradigm, which was suggested by Wemple and Di Domenico, as indicated in Equation (12) [45]:

(12) $$\frac{1}{n^{2}-1}=\frac{E_{O}}{E_{d}}-\frac{1}{E_{O}\;E_{d}}{\left(h\nu \right)}^{2}$$

where *E_a_* represents the single-oscillator energy, and *E_d_* refers to the dispersion energy, which measures the intensity of inter-band transitions and the variations correlating with the structural arrangement of the material. Therefore, by plotting the relation between (n^2^ − 1)^−1^ and ${\left(hv\right)}^{2}$ of the pristine MC, MC/PANI, and MC/PANI/AgNPs films as displayed in Figure 8a, we can deduce the values of the parameters *E_a_* and *E_d_* through the intercept of the y-axe and the slope of the linear fit part, respectively. Additionally, the static refractive index values (n*_o_*) of the pristine MC, MC/PANI, and MC/PANI/Ag films can be computed via extrapolating the upright portion of each curve with the ordinate ${\left(hv\right)}^{2}\;$= 0 as shown in Equation (13) [46]:

(13) $$n_{o}={\left(1+\frac{E_{d}}{E_{O}}\right)}^{1/2}$$

The zero frequency of the dielectric constants (ε_∞_) of the films was determined by employing the relation ε∞ = (*n*_o_)^2^. The parameters E_o_, E_d_, and ε_∞_ for the pristine MC, MC/PANI, and MC/PANI/Ag films were estimated and are recorded in Table 2. As noticed, the enhancement of dispersion energy from 0.43 for pure MC to 0.87 for MC/3%PANI and to 0.90 for MC/3%PANI/1%AgNPs implies that the electronic structure of MC molecules suffered from both the modifications of PANI and AgNPs added to MC, and the defects developed in the MC matrix.

Besides, other parameters such as the lattice dielectric constant (*ε_l_*) and the ratio concentration of free carrier and its effective mass (N/m*) can be deduced using another relation of the real part of dielectric constant in the Spitzer–Fan model (Equation (14)) [47]:

(14) $$\varepsilon_{r}=\varepsilon_{l}-\left(\frac{e^{2}}{4\;\pi^{2}\varepsilon_{s}c^{2}\;}\frac{N}{m^{*}}\right)\lambda^{2}$$

where *c* represents the light speed, *e* defines the charge of an electron and *ε_s_* demonstrates the free space dielectric. Therefore, by plotting the relevance among the real part dielectric constant and λ^2^ for the pristine MC, MC/PANI, and MC/PANI/AgNPs films as displayed in Figure 8b. The values of *ε_l_* and N/m* are obtained from the intercept of x-axe and the slope of the straight portions, respectively. Furthermore, the plasma resonance frequency values (W*_p_*) for each valence electron included in the optical transitions is estimated by employing the following equation (Equation (15)) [48]:

(15) $$W_{p}=\frac{e^{2}}{\varepsilon_{o}}\times \frac{N}{m^{*}}\;$$

As noticed from the data of Table 2, the values of *ε_l_*, N/m* and W*_p_* increased with the increase in the contents of both PANI and AgNPs. These increases have been attributed to the variation in bond length with the addition of PANI and AgNPs. In particular, ε∞ and N/m* data reduced with the increase in the AgNPs content due to the decline in the number of dipoles contributed in the polarization.

The long-wavelength refractive index (n∞) and medium oscillator-wave longitude (λo) of the pristine MC, MC/1%PANI, MC/3%PANI, MC/3%PANI/0.5%AgNPs and MC/3%PANI/1%AgNPs films are determined by analyzing the data of the refractive index utilizing the Sellmeier-oscillator equation (Equation (16) [49]:

(16) $$(n_{\infty }^{2}-1)/\left(n^{2}-1\right)=1-{\left(\frac{\lambda_{o}}{\lambda }\right)}^{2}$$

By studying the relation between (*n*^2^ − 1)^−1^ and λ^−2^ at longer wavelengths as shown in Figure 8c, the values of *n*_∞_ and *λ_o_* can be obtained from the intercept of the x-axe and the slope of the linear part of the detour, respectively. It is clear from the data of Table 3 that the value of *n*∞ and *λ* increases with the increase in both PANI and AgNPs. The *λ_o_* increased from 477 nm to 500 nm, while the *n*_o_ increased from 1.11 to 1.26 when the silver content changed from 0.5% to 1%. Specifically, by hiring the single Sellmeier-oscillator at depressed energy (*n*_o_^2^ − 1/*n*^2^)^−1^ = 1 − (λ_o_/λ)^2^, it was possible to compute the value of oscillator length intensity (So) through the following equation (Equation (17)) [50]:

(17) $$S_{o}=(n_{\infty }^{2}-1)/{\left(\lambda_{o}\right)}^{2}\;$$

In the Drude model, the imaginary part of dielectric constant *ε_i_* relies on the energy of the incident photon and is associated with the incident photon wavelength using Equation (18) [51]:

(18) $${\;\varepsilon }_{i}=\frac{1}{4\pi^{3}\varepsilon_{o}}\left(\frac{e^{2}N}{c^{3}m^{*}\tau }\right)\lambda^{3}$$

Therefore, the relaxation time values (**τ**) of all the fabricated films can be determined through the plotting of the *ε_i_* and λ^3^, as shown in Figure 8d, and listed in Table 3. As can be seen, the time relaxation values gradually decreased with an increase in both the PANI and AgNPs. It reduced from 8.8 × 1014 sec for pure MC to 5.7 × 1014 sec for MC/3%PANI and to 1.1 × 1014 sec for MC/3%PANI/1%AgNPs. These results confirmed that the formed films have become more convenient to high rapid optoelectronic devices [52]. 

When light waves pass inside the polymer, they interact with the charge particle of that polymer, producing a dipole movement which provides the non-linear electron polarizability (*P*). Accordingly, the composite films possess an optical nonlinearity (NLO) and rapid response time. In this study, the NLO of the fabricated films can be featured by Equation (19) [53]:

(19) $$P=X^{\left(1\right)}E+X^{\left(2\right)}E^{2}+X^{\left(3\right)}E^{3}$$

where *P* defines polarization of the material,$\;X^{\left(1\right)}$ represents the linear susceptibility,$\;X^{\left(2\right)}$ relates to the second order NLO susceptibility which corresponds to a second harmonic generation, and 𝑋^(3)^ refers to the third order NLO susceptibility. Thus, it was possible to calculate the values of $\;X^{\left(1\right)}$ and $X^{\left(3\right)}$ by using the following relations (Equations (20) and (21)) [54]:

(20) $$X^{\left(1\right)}=\frac{\left(n^{2}-1\right)}{4\pi }$$

(21) $$X^{\left(3\right)}=A{\left(X^{\left(1\right)}\right)}^{4}\;$$

The refractive index can thus be written in the following form [50].

(22) $$n\left(\lambda \right)=n_{o}\left(\lambda \right)+n_{2}\left(E^{2}\right)$$

**Figure 9:**
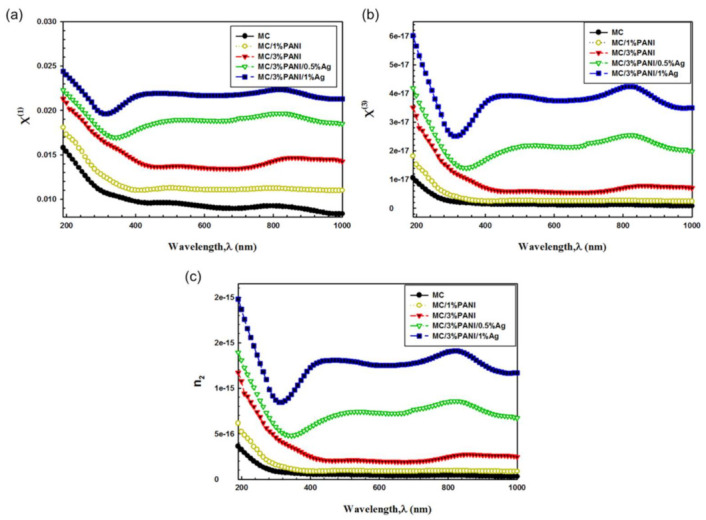
(**a**) linear optical susceptibility $X^{\left(1\right)}$, (**b**) non-linear optical susceptibility $X^{\left(3\right)}$ and (**c**) non-linear refractive index n_2_; as a function of wavelength λ for pristine MC, MC/1%PANI, MC/3%PANI, MC/3%PANI/0.5%AgNPs and MC/3%PANI/1%AgNPs films.

*n_o_* refers to a linear refractive index, and *n*_2_ represents the NLO refractive index. The values of the NLO refractive index from the NLO susceptibility and refractive index (*n*) can be extracted from the following relation [54]:

(23) $$n_{2}=\frac{12\pi X^{\left(3\right)}}{n_{o}}$$

where, *n_o_* = *n*, because n_o_ >> *n_2_*. The change of $X^{\left(1\right)}$ and $X^{\left(3\right)}\;$ versus wavelength (λ) are depicted in Figure 9a,b, respectively, of pristine MC, MC/1%PANI, MC/3%PANI, MC/3%PANI/0.5AgNPs and MC/3%PANI/1%AgNPs films. Notably, the values of $X^{\left(1\right)}$ and $X^{\left(3\right)}$ increased with an increase in both the PANI and AgNPs contents. This increase was due to the creation of defect centers, which led to a boost in local polarizability. Moreover, the increase in $X^{\left(1\right)}\;$ and $\;X^{\left(3\right)}$ with the increase in both PANI and AgNPs was attributed to the enhancement in the interaction of AgNPs with the PANI/MC blend [55]. This result showed the advantages of fabrication composite films composed of PANI and AgNPs, which are envisioned to enhance the absorption for the optical devices. 

The spectral position and intensity of plasmon resonance strongly depends on the organization of metallic NPs and the properties of the surrounding dielectric matrix [56]. Therefore, by changing the concentration of the metal nanoparticles and structure modification of polymer matrix, the optical properties will change [57]. In the other words, charge carriers from the continuous network inside the composite films can modify the linear and non-linear optical behavior [58]. Figure 9c depicts the change of the non-linear refractive index against the wavelength of the pristine MC, MC/1%PANI, MC/3%PANI, MC/3%PANI/0.5AgNPs and MC/3%PANI/1AgNPs films. Notably, the values of *n_2_* have the same trend of the valued of χ^(3)^, as it gradually increased with the increase in both the PANI and AgNPs content. These results clearly showed the importance of adding the conducting PANI and the AgNPs to MC where the resultant films indicted an improvement in the nonlinear optical parameters, which is favored for the optoelectronic device applications [59].

## 4. Conclusions

MC/PANI blends and MC/PANI/AgNPs composite films were fabricated using the casting method and successfully characterized by different spectroscopic techniques. The linear/nonlinear optical parameters of MC/PANI/AgNPs composite films were determined using the UV–Vis spectroscopy. The XRD results showed successful fabrications of MC/PANI/AgNPs composite films, with AgNPs crystallite size of 13 nm. The optical energy band gap of the MC composite film decreased by the addition of both the PANI and AgNPs. The optical conductivity of the prepared MC-based films increased by doping of PANI and AgNPs. By changing the AgNPs content from 0.5% to 1%, the motion of charge carriers and localized charged particles fluctuations produced modifications in the optical dielectric dispersion properties. The results indicate that the prepared films can be considered as promising flexible materials for optical energy applications.

## Figures and Tables

**Table 1:** Values of absorption edge (Ed), band gap (*E*_g_), band tail (*E*_e_), and carbon cluster number N of pristine MC, MC/1%PANI, MC/3%PANI, MC/3%PANI/0.5%AgNPs and MC/3%PANI/1%AgNPs films.

The Samples	Absorption Edge (*E*_d_) (eV)	Optical Band Gap (*E*_g_) (eV)	Urbach Energy (*E*_e_) (eV)	Carbon Cluster Number (N)
Pure MC	5.42	5.76	0.51	36
MC/1%PANI	3.70	5.06	1.38	45
MC/3%PANI	3.14	5.01	1.52	47
MC/3%PANI/0.5%Ag	2.38	4.41	1.92	61
MC/3%PANI/1%Ag	1.93	4.27	2.02	65

**Table 2:** Values of the optical parameters *E*_o_, *E*_d_, ε_∞_, ε_l_, N/m* and W_p_ of the pristine MC, MC/1%PANI, MC/3%PANI, MC/3%PANI/0.5AgNPs and MC/3%PANI/1AgNPs films.

The Samples	*E*_o_ (eV)	*E*_d_ (eV)	ε_∞_	ε_l_	N/m* × 10^38^ Kg^−1^ m^−3^	W_p_ × 10^12^ (S^−1^)
Pure MC	4.1	0.43	1.10	1.11	0.40	0.116
MC/1%PANI	5.12	0.74	1.12	1.14	0.25	0.072
MC/3%PANI	4.80	0.87	1.18	1.18	0.20	0.058
MC/3%PANI/0.5%AgNPs	3.87	0.90	1.23	1.25	0.60	0.171
MC/3%PANI/1%AgNPs	4.40	0.90	1.19	1.28	0.50	0.150

**Table 3:** Values of the optical parameters Wp, *n*∞, λo, So, and **τ** of the pristine MC, MC/1%PANI, MC/3%PANI, MC/3%PANI/0.5Ag and MC/3%PANI/1Ag films.

The Films	*n_o_*	*λ_o_* (nm)	*S_o_* × 10^12^ (m^−2^)	τ × 10^−14^ (s)
Pure MC	1.05	412	0.58	8.8
MC/1%PANI	1.07	244	2.38	6.6
MC/3%PANI	1.18	346	3.20	5.7
MC/3%PANI/0.5%Ag	1.11	477	1.02	1.4
MC/3%PANI/1%Ag	1.26	500	2.35	1.1

## Data Availability

The data of this work are available upon request from Mohamed R. Berber and A. Atta.
